# Atypical Teratoid/Rhabdoid Tumor Originated From the Trigeminal Nerve in a Young Male Adult: Case Report and Review of the Literature

**DOI:** 10.3389/fneur.2020.00265

**Published:** 2020-04-21

**Authors:** Fuxiang Chen, Wenzhong Mei, Wen Lu, Tiefa Zeng, Dezhi Kang, Xiyue Wu, Honghai You

**Affiliations:** ^1^Department of Neurosurgery, The First Affiliated Hospital of Fujian Medical University, Fuzhou, China; ^2^Department of Disease Prevention and Healthcare, Fujian Provincial Hospital, Fuzhou, China

**Keywords:** atypical teratoid/rhabdoid tumor, adult, trigeminal nerve, cranial nerve, surgery

## Abstract

Atypical teratoid/rhabdoid tumor (AT/RT) is a highly malignant central nervous system neoplasm predominantly found in children under the age of 3 years, and is extremely rare in adults. There is no specific clinical presentations or radiological features in reported cases of AT/RT. Diagnosis of brain AT/RT is mainly dependent on the classical pathological characteristics. We report a rare case of AT/RT arising from the trigeminal nerve and leading to progressively multiple cranial nerve palsies in a 25-year-old male patient. Microsurgical resection of the tumor has been performed and confirmed the diagnosis by postoperative pathology. To our knowledge, this is the second case of adult-onset AT/RT originating from the trigeminal nerve.

## Introduction

Atypical teratoid/rhabdoid tumor (AT/RT) is a highly malignant tumor of the central nervous system (CNS), that is mostly discovered in infancy and young children, and is extremely rare in adults (1). It was firstly named by Rorke et al. (2) and was defined to be a grade IV tumor according to the 2016 World Health Organization classification of CNS tumors (3). Combination treatment of surgical resection, chemotherapy, and radiation therapy is currently widely used. However, the overall prognosis of AT/RT is still very unsatisfactory.

AT/RT distinctively presents with the deactivation of integrase interactor 1 (INI-1) tumor suppressor gene, also known as hSNF5/SMARCB1, which is located on chromosome 22q11.2. It has been widely accepted that mutation of INI-1gene or lack of protein expression plays a decisive role in the diagnosis of AT/RT (4, 5). The clinical manifestations of AT/RT mainly depend on the tumor location. Patients with AT/RT deriving from the cranial nerves have been described in few literature to date (6, 7), but these cases are still rarely reported in adults (8, 9). Here, we report a case of adult-onset AT/RT suspected to be derived from the trigeminal nerve followed by the involvement of multiple cranial nerves in a short period of time. In addition, a review of adult-onset AT/RT arising from the cranial nerves is provided.

## Case Description

**Figure 1:**
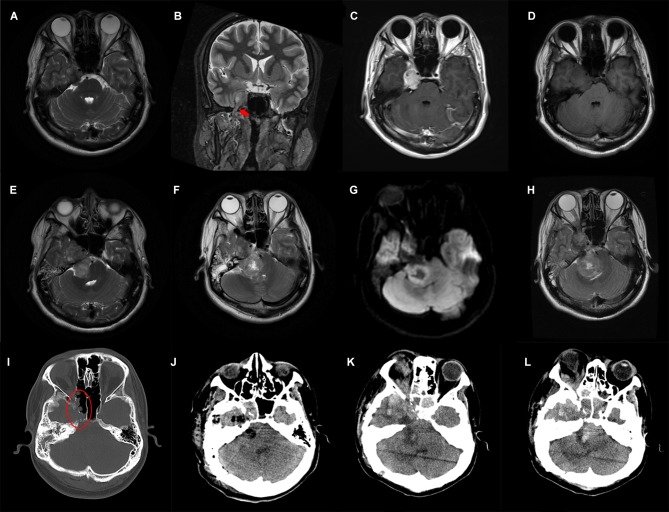
Preoperative and postoperative imaging features of the patient. T2-weighted MRI axial **(A)** and coronal **(B)** images show a solid tumor arising from the trigeminal nerve (red arrow) with heterogeneous contrast-enhanced **(C)**; **(D–H)** Axial MRI images reveal gradual enlargement of the tumor; **(I)** Destruction of the adjacent skull base bone is shown in the oval; **(J)** Postoperative CT shows subtotal removal of the tumor; **(K,L)** Follow-up CT scanned 3 weeks after surgery shows tumor recurrence.

A 25-year-old male with an unremarkable family history presented with a 2-week history of right facial pain, numbness, and double vision. On clinical examination, the young adult was found to be experiencing facial hypesthesia and was unable to abduct the right eye. Cerebrospinal fluid obtained by lumbar puncture was basically normal. Initial magnetic resonance imaging (MRI) of the brain demonstrated a 3.0 × 2.0 cm solid mass originated from the right trigeminal nerve, with heterogeneous contrast enhancement (Figures 1A–C), which was believed to be a trigeminal schwannoma despite less typical imaging features. This patient refused surgical removal of the tumor over concerns about postoperative complications.

A brain MRI a month later showed slightly enlargement of the tumor (Figure 1D), ~4.2 × 2.3 cm in size. The young adult presented with unstable walking, a constant headache, a shallower right nasolabial groove, and contralateral mouth angle droop over the next 2 weeks, which made him readmit to the previous hospital. Subsequently, this patient received a 3-day CyberKnife radiosurgery (DT1800cGy/3f) in short intervals because of his continued rejection of the surgical suggestion. The young man was conscious of the partial alleviation of the above-mentioned symptoms after radiotherapy, while the rescanned MRI (Figure 1E) showed further enlargement of tumor size apart from radiation-induced edema. Emergences of new clinical symptoms, such as right-side hearing loss and ptosis, developed in this male patient in the following 2 months, with tumor extension toward the right cerebellopontine angle (6.3 × 3.2 cm), pushing the brainstem and cerebellum, as well as destruction of the adjacent bones (Figures 1F–I).

The patient was then referred to our institution and surgical removal of the dumbbell-shaped mass was performed on December 2019 via a combination of the suboccipital retrosigmoid approach and the subtemporal approach. The tumor was found to be hard with abundant blood supply and tightly adhered to the surrounding cranial nerves during the operation, resulting in subtotal resection of the mass. There was no significant hemorrhage in the operative field according to the postoperative computed tomography (CT) carried out the next day (Figure 1J). The patient's neurological status remained basically unchanged during the early postoperative period, and he felt relief of his headache and facial pain.

**Figure 2:**
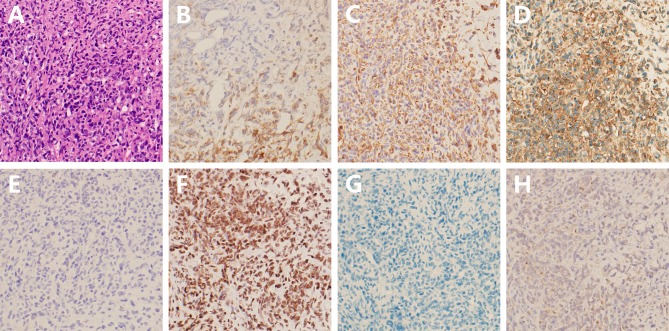
Hematoxylin-eosin and immunohistochemical staining of the tumor tissue. **(A)** Histological staining shows a high cell density with eosinophilic cytoplasm and prominent nucleolus; **(B–F)** Positive expression of EMA, VIM, CD99, synaptophysin, and BRG1, respectively; **(G,H)** Negative expression of desmin and INI-1 (at 200 magnification).

Histologically, the tumor was composed of sheets of cells with eosinophilic cytoplasm, prominent nucleolus, and invasive growth. Parts of the tumor cell appeared with a high nucleocytoplasmic ratio and presented with a blue small cell-like appearance (Figure 2A). Immunohistochemical (IHC) staining revealed partial immunoreactivity for epithelial membrane antigen (EMA), vimentin (VIM), CD99, synaptophysin, smooth-muscle actin (SMA), and brahma-related gene 1 (BRG1, also known as SMARCA4), while desmin, S100, and glial fibrillary acidic protein (GFAP) were negatively expressed in the tumor specimen (Figures 2B–G). In addition, the proliferative index (Ki-67) was 90%. Unexpectedly, loss of expression of the INI-1 protein was discovered in tumor cells (Figure 2H). All these pathological features, especially the lack of INI-1 protein expression, facilitated the diagnosis of AT/RT in this case.

The patient was transferred to the oncology department for his first-stage chemotherapy after surgery recovery, but he suddenly developed respiratory dysfunction on the same day. Instead, treatment for pulmonary infection and respiratory support were performed during those days. Unexpectedly, a brain CT scan 3 weeks after surgery showed an obvious increase in tumor size (Figures 1K–L). He then gave up further treatment and died several days after, out of hospital.

## Discussion

AT/RT has a similar incidence rate in young pediatric patients as medulloblastoma and primitive neuroectodermal tumor (PNET), at approximately 2%, but it is very rare in adults (9, 10). AT/RT occurs more frequently in men than in women, with a ratio of ~2: 1 (8). The patients suffer a poor clinical outcome regardless of their age and gender (11). To date, a total of nearly 55 adult AT/RTs have been reported in the literature (12). Only one of them originated from the trigeminal nerve (9), and this young adult reported here belongs to another one. In the present report, multiple brain nerves were involved successively due to the expansion of the tumor, which then manifested as third to eighth cranial nerve dysfunctions.

Adult-onset AT/RT can occur anywhere in the central nervous system, such as the cerebellar hemispheres, sellar region, spinal cord, pineal region, and cranial nerves (9, 13–16), even secondarily developing from other primary tumors (17, 18). In addition, the clinical manifestations and neuroimaging characteristics of patients often lacked specificity (19). Although mixed intensity in T2-weighted image, heterogeneous contrast enhancement, mild peritumoral edema, high density, and bone destruction on computed tomography have been reported frequently in adult-onset AT/RT (9, 20), there are still great challenges in accurate diagnosis of AT/RT preoperatively. In clinical practice, AT/RT is easily misdiagnosed as other types of intracranial tumor before pathological confirmation. For example, previous reports misdiagnosed cases of AT/RT arising from the optic nerve as the optic pathway glioma because of their strikingly similar characteristics on MRI (7, 21). Two other cases of adult-onset AT/RT spreading along the cranial nerve, together with ours, were also misdiagnosed as schwannomas preoperatively, and even received radiation therapy (Table 1).

**Table 1:** Summary of the adult-onset AT/RT arising from cranial nerves.

**Age**	**Gender**	**Originated from**	**Symptom**	**CT**		**MRI**		**Therapy**	**Expression of INI-1**	**References**
				**Density**	**Bone**	**T2WI**	**Enhancement**			
25	Male	3rd CN	Right facial pain	High	Destruction	Mixed	Heterogeneous	Surgery, radiotherapy	No	/
22	Female	8th CN	Left hearing loss	High	Destruction	Mixed	Heterogeneous	Trimodality	No	(9)
30	Male	3rd CN	Left facial numbness	High	Destruction	Mixed	Heterogeneous	Trimodality	No	(8)

The majority of AT/RTs predominantly contain characteristic rhabdoid cells, with additional neuroepithelial, epithelial, and mesenchymal constituents (22). But aforementioned histopathological features are limited to diagnosis of AT/RT due to these findings, and can also be seen in PNET and medulloblastoma (23). AT/RTs have been reported to exist with 70% mutation or deletion of INI-1 tumor suppressor gene on 22q11.2 (8). However, almost all cases of AT/RT show a loss of expression of INI-1 protein, which has been regarded as a sensitive and specific diagnostic marker (23, 24). As described in Table 1, these three cases of adult-onset AT/RT arising from the cranial nerves were also negative for expression of INI-1 protein. Consistent with previous reports (8, 12), the tumor was also positive for SMA, VIM, CD99, and EMA expression, and negative for desmin, S100, and GFAP expression. Classical immunohistochemical profiles have a high predictive value for differential diagnosis of other brain tumors (8). Notably, the proliferative index was up to 90% in our case, suggesting a high risk of poor clinical outcome.

Multiple therapeutic strategies have been taken for the treatment of pediatric AT/RT (25, 26). Due to the low incidence of AT/RT in adults, the treatment regimen was basically referenced to that of children. Trimodality therapy consisting of maximal tumor resection followed by chemotherapy and radiation therapy is currently the widely accepted treatment (26). Unfortunately, the prognosis of AT/RT is still very poor despite aggressive therapy. Therefore, there is an urgent need for more effective treatments, and exploring pathological mechanisms of adult-onset AT/RT has been one of the key factors. Recent studies have revealed that LIN28B is highly expressed in AT/RT tumors, as well as knockdown of LIN28B inhibited cell proliferation and tumorigenicity. Further studies showed that LIN28B might be regulated through INI-1, suggesting that LIN28B in AT/RT cells might be utilized as a potential therapeutic target (27, 28). In another *in vitro* study, the author found that pretreatment with histone deacetylase inhibitors decreased the proliferation of AT/RT cells and augmented effects of radiation therapy (29). Therefore, further studies toward the pathogenic mechanism of AT/RT may be promising to improve clinical outcomes.

## Conclusion

Adult-onset AT/RT arising from the trigeminal nerve is extremely rare. Initial clinical symptoms depend on the affected cranial nerve. The early diagnosis of AT/RT before surgical resection is very difficult due to its nonspecific imaging features. Patients with AT/RT are still suffering from poor prognosis despite various therapeutic strategies. Understanding of the pathogenesis may provide a new direction for the treatment of adult-onset AT/RT in the future.

## Data Availability Statement

All datasets generated for this study are included in the article/supplementary material.

## Ethics Statement

Written informed consent was obtained from the patient's mother for the publication of any potentially identifiable images or data included in this article.

## Author Contributions

FC and WL drafted the manuscript. WM participated in literature collection. TZ and DK analyzed the MRI features. XW and HY reviewed and revised the manuscript. All authors read and approved the final manuscript.

## Conflict of Interest

The authors declare that the research was conducted in the absence of any commercial or financial relationships that could be construed as a potential conflict of interest.
